# Pericardial Mesothelioma in a Dog: The Feasibility of Ultrasonography in Monitoring Tumor Progression

**DOI:** 10.3389/fvets.2019.00121

**Published:** 2019-04-18

**Authors:** Rina Nabeta, Yuki Nakagawa, Shiori Chiba, Hou Xiantao, Tatsuya Usui, Kazuhiko Suzuki, Tetsuya Furuya, Ryuji Fukushima, Tsuyoshi Uchide

**Affiliations:** ^1^Laboratory of Veterinary Surgery, Tokyo University of Agriculture and Technology, Fuchu, Japan; ^2^Department of Pet Science and Technology, Shandong Vocational Animal Science and Veterinary College, Weifang, China; ^3^Laboratory of Veterinary Pharmacology, Tokyo University of Agriculture and Technology, Fuchu, Japan; ^4^Laboratory of Veterinary Toxicology, Tokyo University of Agriculture and Technology, Fuchu, Japan; ^5^Laboratory of Veterinary Microbiology, Tokyo University of Agriculture and Technology, Fuchu, Japan; ^6^Animal Medical Center, Tokyo University of Agriculture and Technology, Fuchu, Japan

**Keywords:** early detection, dog, pericardial mesothelioma, pleural dissemination, ultrasonography

## Abstract

A 6-year-old neutered male Yorkshire Terrier presented with recurrent pericardial effusion. Although clinical examinations including computed tomography were inconclusive, an exploratory thoracotomy revealed multiple small nodules and plaques on the inner surface of the pericardial sac (Day 1). A subtotal pericardiectomy was performed to prevent cardiac tamponade due to the increasing pericardial effusion, and the resected section of the pericardium was histopathologically diagnosed with mesothelioma. After surgery, chemotherapy with intrathoracic carboplatin was commenced. During the course of the treatment, a detailed follow-up ultrasonographic scan was performed to detect early lesions disseminated on the pleura, originating from the primary pericardial mesothelioma. On Day 101, the minute pleural nodules, which were disseminated lesions as predicted, were successfully imaged by ultrasonography. As the clinical stage advanced, the nodules were observed to gradually increase in size and number, implying tumor progression. These observations highlight the feasibility of ultrasonography in detecting minute disseminated lesions at an early stage, monitoring tumor progression, and thereby, predicting the prognosis of canine pericardial mesothelioma.

## Background

Mesothelioma is a tumor of mesodermal origin that arises from the mesothelium, a monolayer of flattened epithelial cells lining the surface of the pericardial, pleural, and peritoneal cavities including the tunica vaginalis of the testes (1–3). This tumor of the serous membrane is rare in dogs, representing approximately 0.2% of all canine tumors (4). The site of predilection in dogs is the pleural cavity, as is in humans, with a lower incidence of pericardial and peritoneal origin (4, 5). The major clinical signs of thoracic mesothelioma are recalcitrant pleural and pericardial effusion leading to respiratory distress and cardiac tamponade (2, 3, 5). In order to improve the therapeutic outcomes of this tumor, it is important to control pleural and pericardial effusion as well as tumor progression through careful monitoring. Although ultrasonography has been commonly used for the follow-up monitoring of effusion, it has not been proven that the modality can assess tumor progression via the detection of newly disseminated minute lesions (6–8). Here we report a case of pericardial mesothelioma in a dog in which ultrasonography successfully detected the minute tumor lesions disseminated on the pleura during the course of tumor progression.

## Case Presentation

A neutered, male, 6-year-old Yorkshire Terrier weighing 1.76 kg was referred to the Animal Medical Center (AMC) at the Tokyo University of Agriculture and Technology with pericardial effusion of modified transudate. Recurrent effusion could not be controlled with standard treatment including antibiotics and steroids at a primary veterinary clinic.

## Laboratory Investigations and Diagnostic Tests

At AMC, in order to detect the underlying cause of fluid production, detailed examinations, including physical examination, sphygmomanometry, complete blood count (CBC), plasma biochemical analysis, as well as radiography, and ultrasonography were performed. Physical examination was unremarkable. Blood pressure was relatively high on sphygmomanometry (systolic blood pressure: 160 mmHg; diastolic blood pressure: 104 mmHg; mean arterial pressure: 125 mmHg; reference range: systolic blood pressure: 133 mmHg; diastolic blood pressure: 75.5 mmHg; mean arterial pressure: 98.6 mmHg) (9). The CBC revealed mild hyperchrome (mean corpuscular hemoglobin concentration: 36.5 g/dl; reference range: 32–36 g/dl) and mild thrombocytosis (platelet count: 60.3 × 10^4^ μl; reference range: 20–50 × 10^4^ μl), and the plasma biochemical profile revealed mild azotemia (blood urea nitrogen: 30.9 mg/dl; reference range: 9.2–29.2 mg/dl) and moderately elevated levels of alanine transaminase (120 U/l; reference range: 17–78 U/l). White blood cell count, plasma C-reactive protein, and albumin were within respective reference ranges. Serum cardiac troponin I (IDEXX Laboratories, Tokyo, Japan) was also within normal range. From these results, inflammatory diseases, hypoalbuminemia, and uremia were unlikely to be the cause for effusion. The non-specific results of the blood tests warranted a more detailed examination by diagnostic imaging to search for the presence of any cardiovascular disorders or neoplastic diseases.

Ultrasonography detected a small to moderate amount of pericardial and pleural effusion. However, despite the more careful scanning of the thorax and abdomen, no significant findings suggestive of cardiovascular disorders or neoplastic diseases causing pericardial and pleural effusion were obtained. Next, computed tomography (CT) imaging was performed under general anesthesia to exclude the possibility of underlying neoplastic disease within the overall pleural cavity. While CT images confirmed bilateral small to moderate fluid accumulation within the thoracic and pericardial cavities, there was no confirmatory evidence of malignancy as reported previously (10). For the purpose of preventing subsequent cardiac tamponade from the increasing pericardial effusion, and uncovering the underlying cause for the effusion, a subtotal pericardiectomy and macroscopic observation of the entire pleural and pericardial cavities was performed (Day 1).

**Figure 1:**
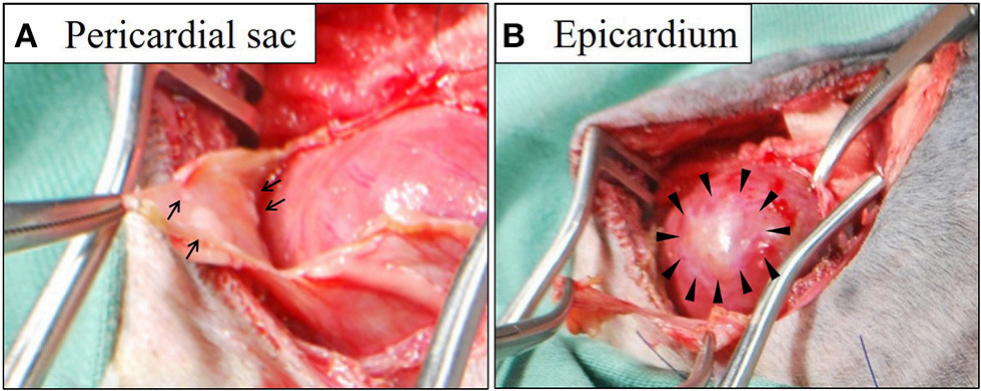
Gross findings of the pericardial sac **(A)** and epicardium **(B)** during surgery. Multiple white nodules (arrows in **A**) and plaques (arrowheads in **B**) were identified on the surface of the thickened pericardial sac and the epicardium, respectively.

**Figure 2:**
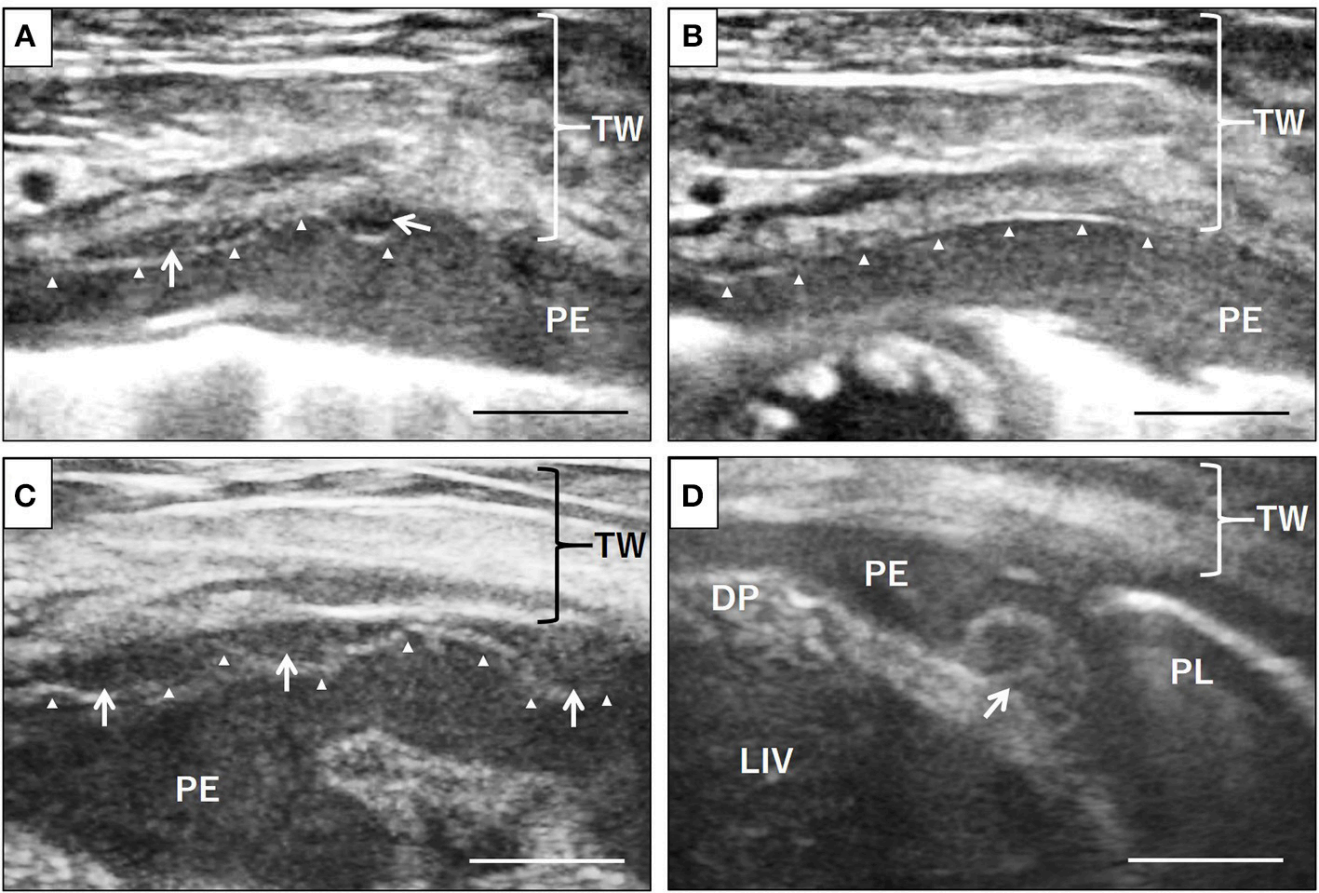
Ultrasonographic evaluation of disseminated neoplastic lesions on the pleura. Small nodules of 1-1.3 mm size on the surface of the pleura, exhibiting homogeneous low echogenicity inside with a hypoechoic structure, were identified, which had not been noted at the early stage of the disease, via detailed ultrasonographic examination using a high frequency linear probe (**A**, arrows). The adjacent normal pleurae were delineated as finely hyperechoic linear structures (**B**, arrowheads). The nodules gradually increased in size and number on the parietal pleura (**C**, arrows) as well as the visceral pleura and diaphragm (**D**, arrows). Arrowheads, parietal pleura; TW, thoracic wall; PE, pleural effusion; DP, diaphragm; PL, lung; LIV, liver. Scale bar = 0.5 cm.

During surgery, in addition to serosanguinous pleural effusion; multiple white, minute-to-small nodules and plaque lesions were observed on the inner surface of the pericardial sac (Figure 1). The outer surface of the pericardial sac and overall serous membrane within the pleural cavity was found intact under careful intraoperative observation. The pleural effusion, on subsequent cytomorphological examination and bacterial culture, was reported as an exudate containing hyperplastic, reactive mesothelial cells with no infectious agents. Histopathological examination of the resected pericardial sac confirmed mesothelioma. Because the mesothelioma lesions were identified only on the serous pericardia (i.e., the inner surface of the pericardial sac), a diagnosis of primary pericardial mesothelioma was made. Since further cytological examination of the pleural fluid including the immunocytochemical differentiation of reactive hyperplasia from malignant neoplasia was not performed, the possibility of the presence of early microscopic lesions on the pleura disseminating from the primary site could not be excluded. However, conclusive proof of subsequent severe metastasis of the tumor cells into the thoracic cavity from the pericardial cavity was certainly predicted due to the surgical procedure, and the aggressive nature of the mesothelioma. Therefore, careful monitoring by detailed ultrasonographic scanning throughout the thoracic cavity using a high frequency linear probe (10 MHz, Hitachi, Ltd, Japan) was performed on follow-up to capture early, disseminated cancerous lesions on the pleura. During the course of monitoring the scanning operation was conducted by one skilled physician in order to ensure validity. Follow-up chest ultrasonography revealed recurrent pleural effusion on Day 38, and successfully detected multiple minute nodules 1.0–1.3 mm in size, which were early disseminated lesions on the parietal pleura, on Day 101 (Figure 2A). The nodules were dome-like or plaque-like in shape and adhered to the surface of the parietal pleura, exhibiting homogeneous low echogenicity inside with a hyper-echogenic linear structure on the surface. The margins of the nodules were smooth and extended along the pleura. The adjacent intact pleurae were delineated as fine, hyperechoic linear structures (Figure 2B).

For a precise follow-up examination, especially for the evaluation of size and number of nodules, scanning was always performed in the standing position, and the scanning window was set on the right ventral region of the second intercostal space, where the minute lesion was initially identified. As the disease progressed, the nodules gradually increased in size and number regardless of the intermittent resolution of the pleural effusion by chemotherapy. At the advanced stage, the nodules coalesced with each other and covered the surface of the pleura, leading to pleural thickening (Figure 2C). Scanning other regions of the chest wall identified similar nodules on the diaphragm (Figure 2D) and the parietal pleura of other ventral regions. The nodules acquired by biopsy were pathologically confirmed as disseminated lesions of pericardial mesothelioma. As treatment, in addition to thoracentesis, intrathoracic chemotherapy with carboplatin at a dose of 100 mg/m^2^, and gemcitabine at a dose of 2 mg/kg weekly was administered for 6 months. The dog died of progressive respiratory distress seven and a half months after the surgery; an autopsy was not performed as the owner opted against it.

## Discussion

In the management of thoracic mesothelioma, the evaluation of therapeutic effects in controlling the pleural and pericardial effusion as well as tumor progression is important. While the effusion may be easily detected by ultrasonography, precise assessment of tumor progression, particularly at an early stage, has never been conducted by ultrasonography, since the imaging modality often fails to identify minute lesions that grow and spread invasively along the thin layer of the mesothelium in the body cavity (2, 3, 5). Indeed, to the best of our knowledge, no report on the early detection of such minute disseminated lesions by this modality is available. However, in this case, detailed and careful high-magnification scanning with a high frequency linear probe identified new lesions as minute as 1 mm on the parietal pleura, which were pathologically diagnosed as disseminated lesions from the primary pericardial tumor. Notably, during the course of monitoring, the nodules gradually increased in size and number, implying tumor progression. Concurrent with the tumor progression on ultrasonography, the clinical condition of the dog, particularly its respiratory status, became progressively worse. In fact, even after removal of the pleural effusion, no satisfactory improvement of the respiratory status was observed at the later stage. Based on the fact that parietal pleural thickening and pulmonary atelectasis were becoming more severe, as observed on ultrasonography, this clinical deterioration could have resulted from insufficient expansion of the lung lobe, presumably due to pleural sclerosis. In accordance with ultrasonographic findings and clinical status, it was concluded that ultrasonography plays a pivotal role in the early detection and monitoring, as well as prognosis prediction of this tumor. In contrast, the amount of pleural effusion did not correlate with the tumor progression on ultrasonography, as there was a transient resolution of the effusion while the neoplastic nodules increased in size and number. Determination of the tumor stage based on the presence of the pleural effusion could mislead the practitioners to an inappropriate choice of treatment and incorrect prediction of prognosis.

To detect the minute tumor lesions on the parietal pleura, the presence of pleural effusion was important, to create an echo-free space between the parietal and visceral pleurae, thus providing a good contrast to the mass against the surrounding area. However, in the absence of pleural effusion, with the parietal and visceral pleurae in close contact, detection of the early minute lesions on the pleura was not possible due to the strong reflection on the surface of the lung rich in air. For the evaluation of tumor progression, detailed scanning of the parietal pleura by ultrasonography before the complete drainage of the fluid could improve the chances of detecting early minute lesions.

In this case, the disseminated tumor on the parietal pleura exhibited a characteristic nodular appearance with dome-like or plaque-like shapes with homogeneous, low echogenicity on the inside. Interestingly, this appearance on ultrasonography was similar to that of human mesothelioma of the tunica vaginalis testis, where the hydrocele showed a good contrast against the small nodules attached to the surface of the tunica vaginalis (11). In contrast-enhanced volumetric CT, which is one of the gold standard examinations for mesothelioma in humans, the characteristic findings for malignant pleural diseases including mesothelioma were mainly parietal and nodular pleural thickening (12). In one report of canine thoracic mesothelioma, a characteristic CT finding of parietal subpleural thickening was described (6). In the present case of mesothelioma, ultrasonographic observations during the course of tumor progression revealed that the tumor was growing along the adjacent pleura in an invasive manner. Thus, the tumor progression resulted in pleural thickening resulting from the coalition of minute neoplastic nodules without forming a big neoplastic mass. Given that a recent study on intrathoracic CT features in dogs and cats with pleural effusion described nodules on the pleura as a good predictor for malignancy (10), the ultrasonographic finding of pleural nodule formation leading to pleural thickening could be beneficial in differentiating mesothelioma from other benign pleural disorders including reactive or inflammatory diseases. Therefore, for cases of dogs presenting with unexplained pleural or/and pericardial effusion, it is recommended that precise ultrasonographic evaluation of the pleura be performed to detect the presence of nodules to improve the value of ultrasonography in diagnosing mesotheliomas.

## Concluding Remarks

In conclusion, this report highlights the feasibility of using ultrasonography in assessment of tumor progression in a case of canine pericardial mesothelioma. Considering that precise evaluation of tumor progression is of vital significance in formulating the therapeutic options, as well as for the prediction of prognosis, non-invasive ultrasonography could be a promising modality to serve the purpose.

## Ethics Statement

This study was carried out in accordance with the recommendations of the Guidelines by the Clinical Research Ethics Committee in the Tokyo University of Agriculture and Technology.

## Consent

Written informed consent for the publication of this case report was obtained from the owner. The report was written retrospectively on a patient treated with appropriate standards of care at a veterinary teaching hospital.

## Author Contributions

RN contributed to writing the manuscript and literature review as well as collecting, interpreting, and describing the imaging findings. YN, SC, and HX assisted by performing the ultrasonography on the patient and collecting the data. T. Usui and KS contributed by collecting and evaluating the pathological data. TF contributed to the molecular pathological discussion and grant-related works. RF performed the described surgery on the patient and confirmed the macroscopic findings. T. Uchide contributed to writing the manuscript along with interpreting, and describing the imaging findings as the professional examiner who performed the ultrasonography on the patient.

### Conflict of Interest Statement

The authors declare that the research was conducted in the absence of any commercial or financial relationships that could be construed as a potential conflict of interest.
